# Successful treatment of infective endocarditis due to pandrug-resistant Klebsiella pneumoniae with ceftazidime-avibactam and aztreonam

**DOI:** 10.1038/s41598-021-89255-8

**Published:** 2021-05-06

**Authors:** Majed F. Alghoribi, Moayad Alqurashi, Liliane Okdah, Bassam Alalwan, Yahya S. AlHebaishi, Abdulmajeed Almalki, Maha A. Alzayer, Abdulrahman A. Alswaji, Michel Doumith, Mazin Barry

**Affiliations:** 1 Infectious Diseases Research Department, King Abdullah International Medical Research Center, Riyadh, Saudi Arabia; 2 King Saud Bin Abdulaziz University for Health Sciences, Riyadh, Saudi Arabia; 3 Department of Pathology and Laboratory Medicine, King Abdulaziz Medical City (KAMC), Ministry of National Guard Health Affairs (MNGHA), Riyadh, Saudi Arabia; 4 Division of Adult Infectious Diseases, Department of Medicine, Prince Sultan Military Medical City, Riyadh, Saudi Arabia; 5 Department of Adult Cardiology, Prince Sultan Cardiac Center, Riyadh, Saudi Arabia; 6 Division of Infectious Diseases, College of Medicine, King Saud University, Riyadh, Saudi Arabia

**Keywords:** Microbiology, Antimicrobials, Bacteria, Clinical microbiology, Infectious-disease diagnostics, Microbial genetics, Pathogens

## Abstract

Pandrug-resistant (PDR) *K. pneumoniae* refractory to conventional treatment has been reported worldwide, causing a huge burden on the healthcare system, patient safety and the economy. *K. pneumoniae* is a prominent opportunistic pathogen causing hospital-acquired and community-acquired infections, but is rarely associated with infective endocarditis. Currently, there are sparse data guiding the optimal regimen when commonly used antibiotics fail, notably for the treatment of endocarditis infections. Here we report our experience in treating a 40-year-old female with PDR *K. pneumoniae* infection of cardiovascular implantable electronic device (CIED) and right-sided infective endocarditis. Initial susceptibility testing of the incriminated pathogen showed an apparent susceptibility to colistin but the prolonged course of colistin, gentamicin and meropenem did not resolve the infection. However, the synergistic combinations of aztreonam with ceftazidime-avibactam was able to overcome resistance and clear the infection rapidly. Genome sequencing showed that the PDR *K. pneumoniae* isolate belongs to the international high-risk clone ST14. The isolate harbored genes encoding NDM-1, OXA-48, CTX-M-14b, SHV-28 and OXA-1, explaining resistance to all β-lactams, including carbapenems. It carried the *armA* gene conferring resistance to all clinically important aminoglycosides and had alterations in GyrA, ParC and MgrB, explaining resistance to ciprofloxacin and colistin.

## Introduction

*Klebsiella pneumoniae* is an important human pathogen responsible for a wide range of severe infections with an increasing scarcity of effective treatments^1,2^. The species is often associated with hospital-acquired bloodstream infections but is rarely a cause of infective endocarditis (IE) or cardiac implantable electronic device (CIED) infection^3–5^. Among gram-negative pathogens, *K. pneumoniae* has been only associated with 1.2% of native-valve IE and 4.1% of prosthetic-valve IE^6^. Currently, there is no clear evidence-based treatment guideline for *K. pneumoniae* causing IE^7^. Hence, most of the reported cases were treated according to the antibiotic susceptibilities of the cultured isolates with or without surgical intervention, which led to successful bacteraemia clearance ranging from 70 to 85% in susceptible strains that are not also hypervirulent (hvKP)^3,6,8–10^.

The emergence of co-resistance to β-lactams, aminoglycosides, quinolones, colistin and tigecycline in *K. pneumoniae* isolates poses a serious therapeutic challenge due to limited treatment options. In recent years, the incidences of pan-drug resistant (PDR) *K. pneumoniae* infections refractory to conventional treatment have been reported globally, causing a significant increase in long-term hospitalizations, morbidity and mortality^11^. Resistance to last-resort carbapenems in this species is mainly mediated by the production of β-lactamases, notably those belonging to the KPC, NDM, VIM and OXA-48-like type enzymes. In Saudi Arabia, *K. pneumoniae* is the most contributing organism to carbapenem resistance among all *Enterobacterales*, increasing from 0 to 33.3% in 10 years (2007–2016)^12^. Resistance to carbapenems in *K. pneumoniae* isolates from Saudi Arabia are mainly associated with the acquisition of OXA-48-like and NDM carbapenemases, although few recent studies have reported the detection of KPC carbapenemases in *Klebsiella* spp. isolates^13,14^.

Combination antibiotic therapy has been used as an option to treat patients with life-threatening PDR *K. pneumoniae* infections^15,16^. However, limited studies showed evidence-based combination antibiotic therapy to treat patients infected by PDR *K. pneumoniae.* This is particularly true for endocarditis, for which treatment options are already limited by the localization and characteristics of the infection. Most of the available information is driven from in vitro studies and just a few numbers of clinical in vivo studies^17^. The recent approach of therapeutic options such as the β-lactam-β-lactamase inhibitors ceftazidime-avibactam (CAZ/AVI) or meropenem-vaborbactam combinations showed potent inhibitors activity against class A and D carbapenemase producers (e.g. KPC, OXA-48-like, GES) but were ineffective against class B carbapenemase producers (e.g. NDM, VIM, IMP)^18^. Clinical trials and in vitro studies have demonstrated the activity of CAZ/AVI against ESBL-, AmpC-, KPC- and OXA-48-producing pathogens^18–21^*.* The combination of CAZ/AVI has been approved to be used as a therapeutic option to treat adults with complicated urinary tract infections, hospital-acquired pneumonia and other infections caused by MDR gram-negative pathogens^22^. Other studies have shown that CAZ-AVI plus aztreonam (ATM) can be an effective therapeutic combination against metallo-β-lactamases (MBLs)^41^. Here, we report the successful treatment of CIED and right-sided IE due to carbapenemase OXA-48- and NDM-producing *K. pneumoniae* strain ST14 using CAZ-AVI plus ATM only.

## Results

### Case record, diagnostic and antibiotic treatment

**Figure 1:**
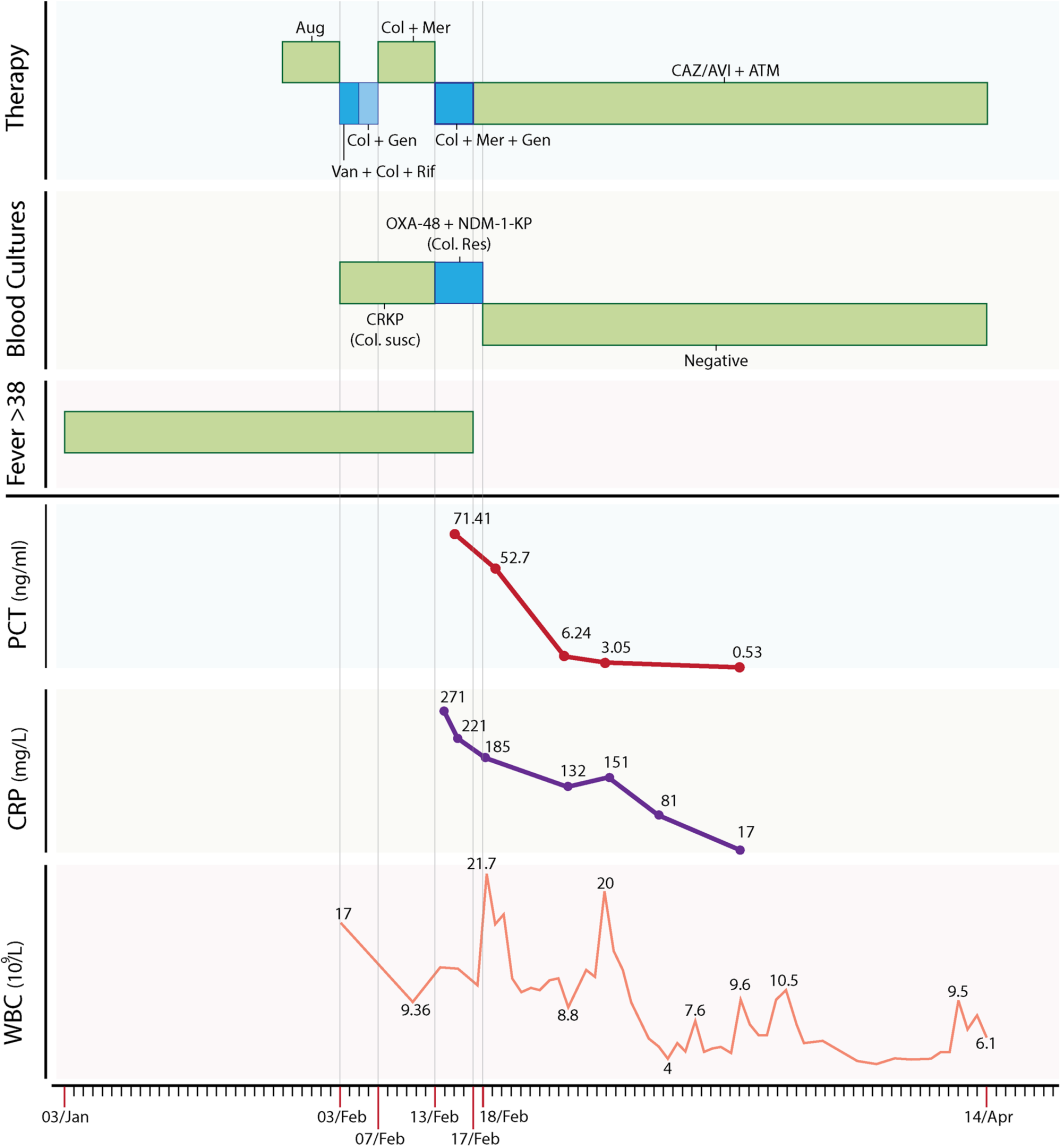
Patient timeline for antimicrobial exposure, fever pattern, duration of persistence of infection in blood cultures with trends of important laboratory investigations. Important dates marked from left to right at the bottom represented the start of symptoms (03/Jan), first hospital admission (03/Feb), transfer to the second hospital (07/Feb), transfer to our cardiac centre (13/Feb), the start of the therapy CAZ/AVI plus ATM combination (17/Feb), date of the first negative culture (18/Feb) and date of discharge from hospital (14/Apr). *Aug* augmentin, *Van* vancomycin, *Col* colistin, *Rif* rifampicin, *Gen* gentamicin, *Mer* meropenem, *CAZ/AVI* ceftazidime/avibactam, *ATM* aztreonam, *CRKP* carbapenem-resistant *Klebsiella pneumoniae*, *OXA-48* oxacillinase, *NDM* New Delhi Metallo-β-lactamase, *Susc* susceptible; *Res* resistant; *PCT* procalcitonin; *CRP* C-reactive protein; *WBC* white blood cells.

The patient is a 40-year-old female with known rheumatic heart disease since childhood who had a bioprosthetic mitral valve replacement in 2015. In July 2017, the patient had the mechanical valve replaced due to valve thrombosis and an insertion of a dual-chamber pacemaker to treat arrhythmia. In October 2017, she developed prosthetic mitral valve endocarditis due to *Enterococcus faecalis* that was cleared by six weeks of treatment with a combination of ampicillin and ceftriaxone. In January 2019, the patient had another infective endocarditis affecting the tricuspid valve, and the pacemaker leads due to a carbapenem-resistant *K. pneumoniae*, for which she was managed during ten days in two different hospitals before being transferred to our specialized cardiac center following several cardiac arrests from septic pulmonary emboli (Fig. 1). The antibiotic susceptibility performed in the transferring hospital suggested apparent susceptibility to gentamicin and colistin, and so, the patient was put on colistin (2.5 million IU twice daily) and gentamicin (7 mg/kg once daily) intravenously (IV), to which 500 mg meropenem twice daily due to acute kidney injury was added at day one. However, repeated blood cultures showed persistent bacteremia with a *K. pneumoniae* strain resistant to all tested antimicrobials, including those used for treatment (Table 1). A synergy of antibiotic combinations showed that only CAZ/AVI with ATM was an effective option for treatment (Table 1). Accordingly, the therapy was changed on day five to CAZ/AVI (2.5 gm every 8 h) and ATM (2 gm every eight hours) IV. Her fever resolved, and bacteremia cleared right after starting this combination, while inflammatory markers and creatinine levels improved over a couple of weeks. At 42 days of therapy, the pacer device and the lead tip were replaced. The culture from the tip of the lead did not grow any bacteria, and consequently, the antibiotic treatment was stopped at day 50 (Fig. 1). Although the source of the bacteremia was not found, the follow up at six months after discharge showed that the patient was completely healthy and asymptomatic.

**Table 1:** Antibiotic resistance in *K. pneumoniae* SA-KpST14.

Antimicrobial category	Antimicrobial agents	MIC (μg/ml) VITEK II	Interpretation (CLSI breakpoints)	Genes associated with resistance
β-lactams	Ampicillin	≥ 32	R	*bla*_OXA-1_, *bla*_SHV-28,_ *bla*_NDM-1,_ *bla*_OXA-48_, *bla*_CTX-M-14b_
	Amoxicillin/Clavulanic acid	≥ 32	R	
	Piperacillin/Tazobactam	≥ 128	R	
	Ceftazidime/Avibactam	> 256*	R	
	Cefoxitin	≥ 64	R	
	Ceftazidime	≥ 64	R	
	Cefepime	≥ 64	R	
	Cefalotin	≥ 64	R	
	Ceftriaxone	≥ 64	R	
	Imipenem	> 32*	R	
	Meropenem	≥ 16	R	
	Aztreonam	> 256*	R	
Fluoroquinolones	Ciprofloxacin	≥ 4	R	*aac(6′)-Ib-cr, gyrA* (S83Y, D87G)*. parC* (S80I)
Aminoglycosides	Amikacin	≥ 64	R	*aac(6′)-Ib-cr, aph(3′)-Ib, aph(6)-Id, dfrA12, armA, aadA2, strAB, aph(3′)-VI*
	Gentamicin	≥ 16	R	
Trimethoprim/ Sulfamethoxazole	Trimethoprim/ Sulfamethoxazole	≥ 4/76	R	*dfrA12*
Polymyxin	Colistin	≥ 64**	R	IS5 disruption of *mgrB* gene
Tetracycline	Tigecycline	≥ 256*	R	*oqxAB*^*§*^, *acrAB*^*§*^
Fosfomycin	Fosfomycin	≥ 1024*	R	*fosA*
Others	Nitrofurantoin	128	R	
Name of the antibiotic combination (ETEST)		**Result of synergy test**		
Meropenem + Tigecycline		No zone		
Meropenem + Ertapenem		No zone		
Meropenem + Fosfomycin		No zone		
Meropenem + Gentamicin		Very small zone of inhibition		
Ceftazidime/Avibactam + Aztreonam		A large zone of Inhibition		

* ETEST method.

**Broth Micro Dilution method.

^§^No genetic evidence was found to infer overexpression.

### Genome sequence analysis

Genome sequencing showed that the carbapenem-resistant *K. pneumoniae* SA-KpST14 isolate harbored five different plasmids (Table 2). In silico analyses identified the strain as sequence type (ST)14 and detected genes explaining resistance to β-lactams, aminoglycosides, quinolones, phenicols and fosfomycin, as shown in Table 1. Otherwise, the genetic disruption of the *mgrB* regulator by insertion sequence IS5 explained resistance to colistin.

**Table 2:** Genetic elements sizes and replicon types of plasmids of SA-KpST14 isolate and the presence of antimicrobial resistance genes.

Named	Size	GC (%)	Plasmid Type	Antimicrobial resistance gene(s)	Accession No
SA-KpST14	5,378,785 bp	57	–	*bla*_OXA-1_, *bla*_SHV-28_, *aac(6′)-Ib-cr*, *catB*, *fosA*	CP071279
pSA-KpST14-NDM-1	269, 329 bp	46	IncHI1B	*bla*_NDM-1_, *aadA2*, *aph(3′)-VI*, *armA, mph(E), msr(E), sul1, dfrA12*	CP071280
pSA-KpST14-OXA48-2	68, 932 bp	51	IncM1	*bla*_OXA-48_, *bla*_CTX-M-14b_, *aph(3′')-Ib, aph(6)-Id*	CP071281
pSA-KpST14-3	166,565 bp	50	IncFIB	–	CP071282
pSA-KpST14-4	20,912 bp	53	IncR	–	CP071283
KpST14-5	2,095 bp	44	–	–	CP071284

**Figure 2:**
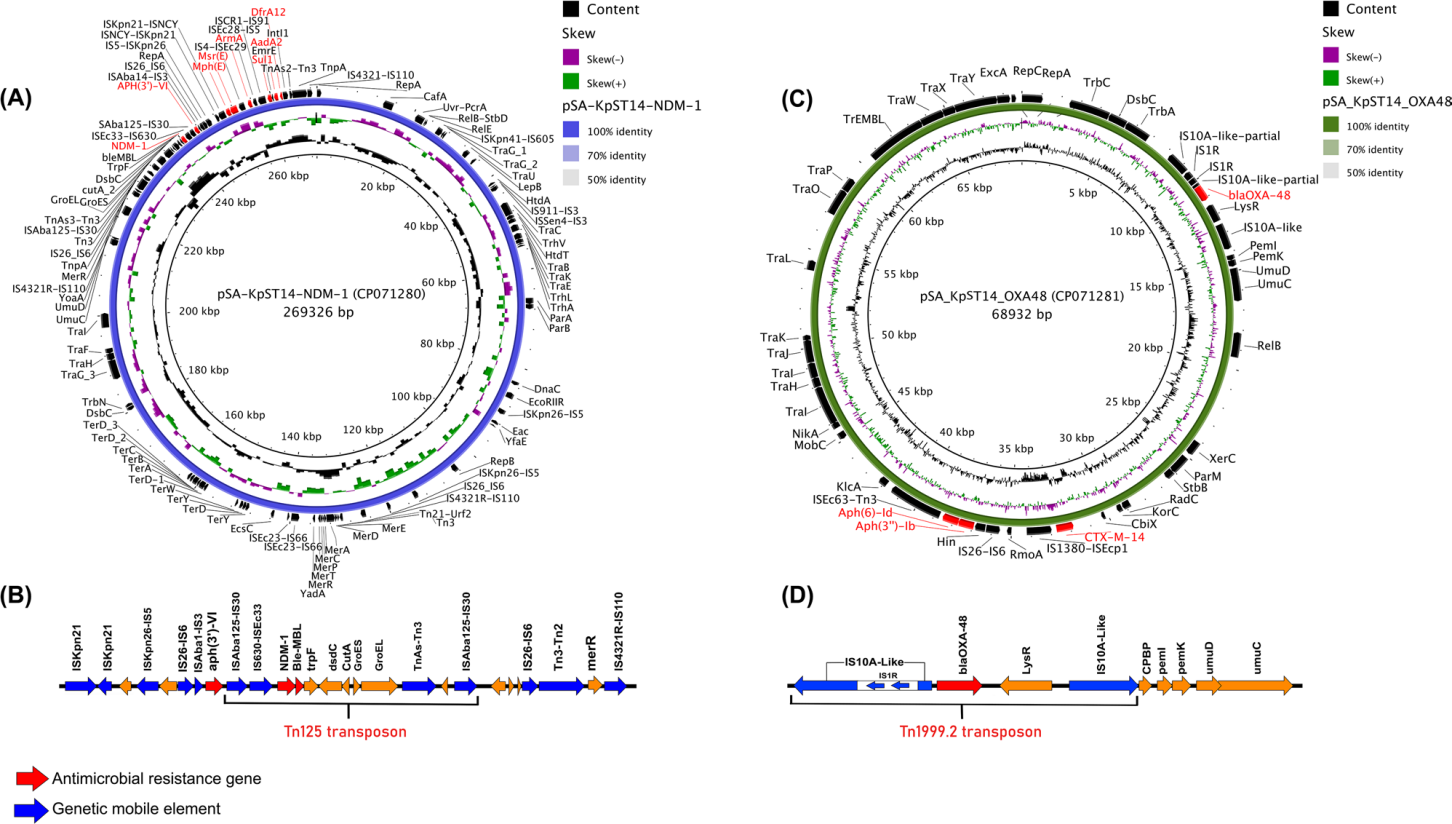
Sequence representation of the two carbapenemase-encoding plasmids carried by the SA-KpST14 isolate. (**A**) Genetic structure of pSA-KpST14-NDM-1 plasmid, (**B**) Gene composition of the bla-_NDM-1_-bearing Tn125 transposon, (**C**) Genetic structure of pSA-KpST14-OXA48-2 plasmid, (**D**) Gene composition of the bla_OXA-48_ -bearing Tn1999.2 transposon. The circular map was generated with the Blast Ring Image Generator (BRIG)^59^ software and the schematic diagram of the genetic structure was generated with Easyfig^60^.

Resistance to last-resort carbapenems was associated with the presence of *bla*_NDM-1_ and *bla*_OXA-48_ genes that were located on two different plasmids (Fig. 2). The *bla*_NDM-1_, embedded in transposon Tn125 (11,192 bp) was located on an IncHI1B replicon-type plasmid (pSA-KpST14-NDM-1, 269, 329 bp) which also harbored resistance to aminoglycosides (*aadA2*, *aph(3′)-VI* and *armA*) , macrolides (*mph(E)* and *msr(E)*)*,* sulfonamides (*sul1*) and trimethoprim (*dfrA12*) (Fig. 2A)*.* The genetic structure of the Tn125 transposon was composed of the insertion sequence ISEc33-IS630 upstream the *bla*_NDM-1_ gene (New Delhi Metallo-beta-lactamase 1) and the *ble*_MBL_ gene (bleomycin resistance protein), *trpF* (phosphoribosyl-anthranilate isomerase), *dsdc* (D-serine deaminase activator), *cutA* (divalent-cation tolerance protein), ATP-dependent chaperonin *GroEL*–*GroES* and incomplete TnAs3-Tn3 downstream (Fig. 2B). On the other hand, the *bla*_OXA-48_ carbapenemase was embedded in a classical Tn1999.2 transposon (5,639 bp) on an IncM1 plasmid (pSA-KpST14-OXA48-2, 68, 932 bp) with also carried the *bla*_CTX-M-14b_ and *aph(3′')-Ib* genes (Fig. 2C). The genetic structure of Tn1999.2 transposon was as described composed of the *lysR* transcriptional regulator, *bla*_OXA-48_ (oxacillinase), flanked by two copies of insertion sequences IS10A-like in the opposite orientation and thus forming the IS10A-like -*bla*_OXA-48_-*LysR*- IS10A-like element. The IS10A-like is belonge to IS4 family which is 99.77% identity with IS10A (Accession number AF078527) and 99.62% identity with IS1999 (Accession number : AF133697).The insertion sequence IS10A-like located upstream of *bla*_OXA-48_ was truncated by the IS1 insertion (Fig. 2D).

### Virulence factors

Genome sequences showed that the *K. pneumoniae* SA-KpST14 strain lacked the capsule regulator gene *rmpA/rmpA2* and the siderophore aerobactin factors that are characteristic of invasive strains. However, the strain possessed several other iron acquisitions and siderophore genes, including the enterobactin (Ent), yersiniabactin (Ybt) and salmochelin (Sal) genes. Of these, the *ybt* locus was identified within the integrative and conjugative element-5 (ICEKp-5) as previously described^23–25^. Capsular type of SA-KpST14 was determined based on the K-locus's gene content, which corresponded to KL2 (99.73%) and known allelic type *wzc*2 *wzi*2. The LPS O antigen was determined by sequence identity to *wzm* and *wzt* genes, which corresponded to O locus O1v1 (99.99%). Otherwise, the isolate harbored other intrinsic virulence factors previously associated with the adherence, biofilm formation, secretion system and efflux pump factors listed in Table 3.

**Table 3:** Virulence factors in *K. pneumoniae* SA-KpST14.

Virulence factor	Category	Related genes	Function
Adherence	Type I fimbriae	*fimA-K*	Adhering to human mucosal or epithelial surfaces
	E. coli common pilus	*ecpRABCDE*	Cell adherence and biofilm formation^26^
Biofilm formation	Type 3 fimbriae	*mrkABCDFHI*	Promotes mucous adherence, tissue colonisation, and biofilm formation^26^
Iron uptake	Enterobactin	*entABCDEF,*	Enterobactin promotes bacterial growth around blood vessels ^27^
		*fepABCDG*	
		*fes*	
		*ybdA*	
	Yersiniabactin	*ybtAEPQSTUX*	Most common virulence genes associated with human *K. pneumoniae* infection ^23–25^
		*fyuA*	
		*irp12*	
	Salmochelin	*iroE*	*iroE* is coded for protein with hydrolytic activity to degrade salmochelins and enterobactin to release iron ^27,28^
Secretion system	T6SS	*tssABCDFGHIJKLM*	Bacterial Competition, Cell invasion, Type-1 fimbriae expression, in vivo Colonization, and to puncture target cells and deliver lethal effectors^29–31^
Immune evasion	K2 capsule	*manBC*	Evading the host immune system ^32^
		*wcaJ*	
		*galf*	
		*gnd*	
		*ugd*	
		*wza, wzi*	
		*cpsACP*	
Serum resistance	LPS	*glf*	Essential structural component and immunodominant molecules of the outer membrane ^33,34^
		*wbbMNO*	
		*wzm wzt*	

## Discussion

Right-sided IE accounts for 5–10% of all IE cases, and more than 50% of these cases are due to intravenous drug use^6^. The presence of an implantable endovascular device imposes a higher risk of developing gram-negative endocarditis along with hospital stay, history of invasive procedures and other risk factors for developing gram-negative bacteremia in general^8^. The weaker adhesion ability of gram-negative bacteria (GNB) has been attributed to the low prevalence of CIED and IE caused by non-HACEK GNB^35^. Presentation can be associated with septic emboli to the lungs as in the presented case^6^. Liver abscess and urinary tract infection are the most common source when bacteremia is present^36^. Mortality rates vary depending on the pathogen, virulence factors, complications and the valve involved, reaching up to 49% in one article^6,37^. Urgent surgical interventions as a first-choice treatment is recommended for uncontrolled infection to prevent complications, including heart failure and embolic events^35,38^.

In *Enterobacterales*, PDR is observed among carbapenemase-producing bacteria, especially among *K. pneumoniae,* as it can easily acquire mobile genetic elements through horizontal gene transfer^39^. The risk of acquiring carbapenemase-producing bacteria increases in a patient with prior surgery, extended hospital stays and the presence of wounds^40^.

Latest Infectious Diseases Society of America (IDSA) guidelines on the treatment of carbapenem-resistant enterobacterales (CREs) in general recommended the use of CAZ/AVI with ATM or cefiderocol monotherapy for MBL-producers (e.g., NDM, VIM or IMP) and CAZ/AVI monotherapy or cefiderocol monotherapies for OXA-48-like producers^41^. While second-line options included tigecycline, eravacycline, colistin and fosfomycin in limited indications and they recommended against combinations of antimicrobials when a β-lactam is susceptible^41^.

However, the guideline did not focus on cases of CIED or IE but rather gave a recommendation for infections outside the urinary tract in general, and no recommendations were given for conditions where two or more resistance genes are detected within the same species^41^.

Therapeutic options used in the literature with successful results for carbapenemase co-producing (NDM-1 and OXA-48 like) *K. pneumoniae* includes the use of ATM in combination with CAZ/AVI as this has shown promising results in NDM producing *Enterobacterales *in-vitro and in-vivo. It is thought that this is due to the efficacy of ATM against MBLs in general with the addition of the effect of the avibactam component in CAZ/AVI on extended-spectrum β-lactamases, and ambler class A, and D carbapenmases, which are often co-produced by some strains^22,42^. The presence of serine β-lactamases along with NDM-1 gene was detected in up to 30% in one study for which the combination of CAZ/AVI and ATM has shown synergy in vitro and in vivo^43^. Several studies have proposed this effect^42,44–47^, and other β-lactamase inhibitors (Clavulanate and Tazobactam) has also been tested with ATM showing variable degrees of successful results^48^. It is worth mentioning that an *in-vitro* study was comparing the synergy of CAZ/AVI plus ATM with Meropenem-Vaborbactam plus ATM in NDM-1 non-OXA-48 like co-producer *E. coli* and *K. pneumoniae* strains which showed similar synergy against these CREs^44^. A single product formulation of aztreonam-avibactam is currently in phase III clinical trial, which will address many issues with using a two-drug combination like susceptibility testing and epidemiologic surveillance data^43^. Clinicians should be aware that resistance to CAZ/AVI in *K. pneumoniae* may emerge while on treatment, and meropenem susceptibility may be restored as previously reported by mutations in the omega loop of *bla*_KPC_ in the carbapenemase-producing strain, which may require testing MICs every time a phenotypic or genotypic alteration occur^49^.

In the presented case, we started the CAZ/AVI combination with ATM on the 4^th^ day of hospital admission, which resulted in rapid clearance of bacteremia (in one day). The decision to start this combination was guided by the available literature at that time (February 2019) and synergy testing using gradient diffusion strips showing positive synergy results. The fact that the patient avoided the indicated open heart surgery for valve replacement just by using this combination proves that it is an effective antimicrobial combination in similar cases.

## Materials and methods

### Bacterial isolate and antimicrobial susceptibility testing

The *K. pneumoniae* strain, namely SA-KpST14, was recovered from a 40-year-old female with rheumatic heart disease (RHD) at Prince Sultan Military Medical City, Riyadh, Saudi Arabia. The initial antimicrobial susceptibility testing was performed using the ﻿VITEK‑2 system (BioMerieux, Brussels, Belgium). Etest (bioMérieux, Durham, NC) was used to determine the MICs for aztreonam, imipenem, ceftazidime/avibactam and synergetic activities of antibiotic combinations listed in Table 1. Antimicrobial MIC interpretations were in accordance to CLSI guidelines. A zone of hope for antimicrobial activity was defined based on the definition of synergy (1 plus 1 equals more than 2); hence, a zone of hope is defined as (0 plus 0 equals more than 1)^50^. MICs of colistin were confirmed by broth dilution methods done according to CLSI guidelines. Carba-R test using GeneXpert system (Cepheid, USA) was initially used to detect the presence of carbapenemase-resistance genes.

### Complete genome sequencing

Genomic DNA (gDNA) of the *K. pneumoniae* SA-KpST14 strain was extracted from an overnight culture on LB agar using the QIAamp DNA Mini Kit (QIAgen, Germany) according to the manufacturer instructions. The quality and purity of the extracted DNA were checked using the Nanodrop 2000 spectrophotometer (Thermofisher, USA) and Qubit 3.0 Fluorometer with the dsDNA HS (High sensitivity) kit (Thermofisher, USA). Short reads sequences were generated on the Illumina MiSeq platform using the Nextera-XT library preparation kit (Illumina, San Diego, CA). Long reads sequencing with the Oxford Nanopore Technology (ONT) were generated using the ligation sequencing kit SQK-LSK109 (Oxford Nanopore Technologies, Ltd., UK) on the MinION sequencer (Oxford Nanopore Technologies, Ltd., UK).

### Hybrid genome assembly and annotation

The Oxford Nanopore MinION and Illumina MiSeq reads were assembled with Unicycler (version 0.4.8)^51^ or following the EToKi pipeline (Enterobase Tool Kit) (version 1.0)^52^ using the default settings. The hybrid genome assembly generated six contigs with an average sequencing coverage depth of 60×. The NCBI Prokaryotic Genome Annotation Pipeline was used for the annotation of the SA-KpST14 chromosome and plasmid sequences^53^. The genome of SA-KpST14 consisted of one 5,378,785 bp chromosome and five plasmids designated pSA_KpST14-NDM-1 (269,329 bp, 46% GC content), pSA_KpST14-OXA48-2 (68,932 bp, 51% GC content), pSA_KpST14-3 (166,565 bp, 50% GC content), pSA_KpST14-4 (20.912 bp, 53% GC content), and pSA_KpST14-5 (2,095 bp, 44% GC content) (Table 2).

### Identification of antibiotic resistance, virulence genes and plasmid replicon typing

Identification of antibiotic resistance genes and virulence factors were determined with ABRicate (https://github.com/tseemann/abricate) (version 0.9.8) using the ResFinder (version 2.1)^54^, Comprehensive Antimicrobial Resistance (CARD)^55^, virulence factors (VFDB)^56^ and Kaptive (version 0.7.3)^57^ databases. Basic plasmid characteristics were determined using the PlasmidFinder (version 1.3)^58^ software. Plasmid maps were drawn using the Blast Ring Image Generator (BRIG)^59^ software and Easyfig^60^.

### Ethical approval

This study was approved by the Cardiac Research Department of Prince Sultan Cardiac Center in Riyadh, Saudi Arabia (Reference number R21004). We confirm that all research in this study was performed in accordance with the relevant guidelines and regulations after obtaining informed consent for conducting and publishing this study. All rules and regulations of ICH-GCP and the Declaration of Helsinki were followed.

### Informed consent

Informed written consent was taken from the patient to conduct and publish this study and no personal data will be disclosed or breached beyond the principal investigator.

## Data Availability

The complete genome sequence of K. pneumoniae isolate SA_KpST14 has been deposited in GenBank under accession no. CP071279 for the chromosome, CP071280 for pSA_KpST14-NDM-1, CP071281 for pSA_KpST14-OXA48-2, CP071282 for pSA_KpST14-3, CP071283 for pSA_KpST14-4, CP071284 for pSA_KpST14-5. These sequences are part of BioProject no. PRJNA705688.
